# Assessment of Heterologous and Homologous Boosting With Inactivated COVID-19 Vaccine at 3 Months Compared With Homologous Boosting of BNT162b2 at 6 Months

**DOI:** 10.1001/jamanetworkopen.2022.26046

**Published:** 2022-08-10

**Authors:** Ee Vien Low, Peter Seah Keng Tok, Masliyana Husin, Jing Lian Suah, Boon Hwa Tng, Thevesh Thevananthan, Maheshwara Rao Appannan, Hazlina Yahaya, Shahanizan Mohd Zin, Faizah Muhamad Zin, Sheamini Sivasampu, Kalaiarasu M. Peariasamy

**Affiliations:** 1 Institute for Clinical Research, National Institutes of Health, Ministry of Health Malaysia, Setia Alam, Malaysia; 2 Disease Control Division, Ministry of Health Malaysia, Putrajaya, Malaysia; 3 Medical Development Division, Ministry of Health Malaysia, Putrajaya, Malaysia

## Abstract

**Importance:**

Evidence for the timing of booster vaccination after completion of BNT162b2 and CoronaVac primary vaccination is crucial to guide policy recommendations.

**Objective:**

To compare the odds of symptomatic SARS-CoV-2 infection and COVID-19–related outcomes after heterologous and homologous boosting of CoronaVac at 3-month intervals and homologous boosting of BNT162b2 at 6-month intervals, with BNT162b2 primary series (2 doses) as the reference group.

**Design, Setting, and Participants:**

This population-based retrospective cohort study used national data for Malaysia. Participants included all individuals aged 18 years and older who received a complete primary series of CoronaVac or BNT162b2 vaccine and were eligible for a booster dose between November 21, 2021, and December 28, 2021. Data were analyzed from November 21, 2021, to January 7, 2022.

**Exposures:**

Receipt of a booster vs no booster and categorized into primary series BNT162b2 (2 doses of BNT162b2), primary series CoronaVac (2 doses of CoronaVac), 3 doses of BNT162b2, primary series CoronaVac plus a BNT162b2 booster, and 3 doses of CoronaVac.

**Main Outcomes and Measures:**

The primary outcome was symptomatic SARS-CoV-2 infection. The secondary outcomes were COVID-19–related intensive care unit admission and death. All outcomes were observed from the day an individual was considered fully boosted (≥14 days after booster dose).

**Results:**

Our cohort included 13 840 240 individuals (mean [SD] age, 39.9 [15.5] years; 7 040 298 [50.9%] men; 4 451 180 individuals [32.2%] with ≥1 comorbidities), of whom 5 081 641 individuals (36.7%) had received a booster dose. Using the primary series BNT162b2 recipients as reference, the adjusted odds against symptomatic SAR-CoV-2 infection were lower for individuals who received the primary series CoronaVac plus a BNT162b2 (adjusted odds ratio [aOR], 0.06 [95% CI, 0.05-0.06]), 3 doses of CoronaVac (aOR, 0.08 [95% CI, 0.06-0.10]), or 3 doses of BNT162b2 (aOR, 0.01 [95% CI, 0.00-0.01]). Receipt of heterologous booster (primary series of CoronaVac plus a BNT162b2 booster) was associated with lower odds of SARS-CoV-2 infection (aOR, 0.17 [95% CI, 0.17-0.18]) compared with homologous booster (3 doses of CoronaVac) for individuals aged 60 years and older (aOR, 0.19 [95% CI, 0.19-0.20]).

**Conclusions and Relevance:**

In this cohort study, for individuals who received the CoronaVac primary series and a booster dose of BNT162b2 or CoronaVac at 3 months, the observed odds of symptomatic SARS-CoV-2 infection were similar to individuals who received the BNT162b2 primary series plus a third dose of BNT162b2 at 6 months. Heterologous booster is recommended for individuals aged 60 years or older who received the CoronaVac primary series, given the lower observed odds against symptomatic SARS-CoV-2 infection among those who received a BNT1612b2 booster.

## Key Points

**Question:**

What are the risks of SARS-CoV-2 infection associated with heterologous and homologous boosting of CoronaVac at 3 months compared with homologous boosting of BNT162b2 at 6 months?

**Findings:**

In this cohort study using national data of 13 840 240 vaccinated individuals, compared with receiving the BNT162b2 primary series, the adjusted risk of symptomatic SARS-CoV-2 infection was lower for heterologous CoronaVac with a BNT162b2 booster and homologous CoronaVac, and 3 doses of BNT162b2 was associated with the lowest risk.

**Meaning:**

These findings suggest that homologous and heterologous boosting of CoronaVac at 3 months after the primary series were comparable with homologous BNT162b2 boosting at 6 months interval in the measure of association against SARS-CoV-2 infection.

## Teaser

This cohort study assesses the risk of symptomatic SARS-CoV-2 infection associated with 2 doses of the CoronaVac vaccine plus a third dose or a BNT162b2 booster compared with 2 or 3 doses of BNT162b2.

## Introduction

Vaccination is crucial to mitigate the effects of COVID-19. However, studies have showed that vaccine-derived protection from currently licensed primary series vaccines wanes substantially over time.

In Israel,^1,2,3,4^ the United States,^5^ and the United Kingdom,^6^ a third dose of mRNA vaccines (BNT162b2 [Pfizer BioNTech] or mRNA-1273 [Moderna]) when administered to individuals who received mRNA primary series vaccination have been found to offer significant protection against severe COVID-19 clinical outcomes. Generally, the timing of homologous boosting for BNT162b2 was at least 5 months after full vaccination (2 doses).^7,8,9,10^

In comparison, for recipients of the inactivated whole-virion SARS-CoV-2 vaccine (CoronaVac [Sinovac Biotech]), there is limited evidence on the optimum timing for a booster dose. Despite immunogenicity studies demonstrating a decline in immune response among recipients of CoronaVac over time,^11,12,13,14^ the World Health Organization (WHO) only recommends a booster dose for high-priority groups (eg, older adults, health care workers, persons with comorbidities) administered 4 to 6 months after the completion of the primary series.^15^

In Malaysia, BNT162b2 and CoronaVac vaccines were highly used in the National COVID-19 Immunization Program (PICK).^16^ Under the PICK rollout, more than 95% of the adult population in Malaysia received their full vaccination by the end of October 2021.^17^ Both BNT162b2 and CoronaVac vaccines were associated with a reduction in SARS-CoV-2 infection, severe COVID-19 (ie, requiring admission into intensive care unit [ICU]), and COVID-19–related deaths,^16^ but the protection waned after 3 to 5 months, especially for recipients of the CoronaVac vaccine.^18^ In Malaysia, a booster vaccination program was initiated from October 13, 2021, at a recommended 6 months after primary series with BNT162b2 and 3 months after primary series with CoronaVac. As of February 6, 2022, 52.8% of Malaysia’s adult population had received their booster doses.^17^

In this retrospective study, we hypothesized that the administration of a booster dose, either homologous (CoronaVac) or heterologous (BNT162b2), at 3 months interval for recipients of the CoronaVac primary series is comparable to homologous boosting at 6 months for recipients of the BNT162b2 primary series. We estimated the odds against symptomatic SARS-CoV-2 infection and COVID-19–related severe outcomes (ICU admission and death) for the booster vaccine compared with the BNT162b2 vaccine primary series across different age groups.

## Methods

This cohort study is part of The Real-World Evaluation of COVID-19 Vaccines under the Malaysia National COVID-19 Immunization Programme study registered in the National Medical Research Register and approved by Medical Research and Ethics Committee, Ministry of Health Malaysia (NMRR-21-1660-60697). As the study was conducted using administrative secondary data, a waiver of participant informed consent was granted. All personal identifier details were removed before computational analysis. This study is reported following the Strengthening the Reporting of Observational Studies in Epidemiology (STROBE) reporting guideline.

### Data Source

We used data sets from Malaysia’s National COVID-19 surveillance system, Ministry of Health Malaysia, including COVID-19 confirmed cases, audited COVID-19 deaths, register of all COVID-19 ICU admissions, register of all vaccination records for the Malaysian population from the Malaysia Vaccine Administration System, list of health care workers eligible for vaccine prioritization, and contact tracing data from the Malaysia’s official contact tracing application (MySejahtera). Data sets were linked using case and national registration numbers. Details of data sources have been described previously.^16^

### Study Design and Study Population

**Figure 1.**
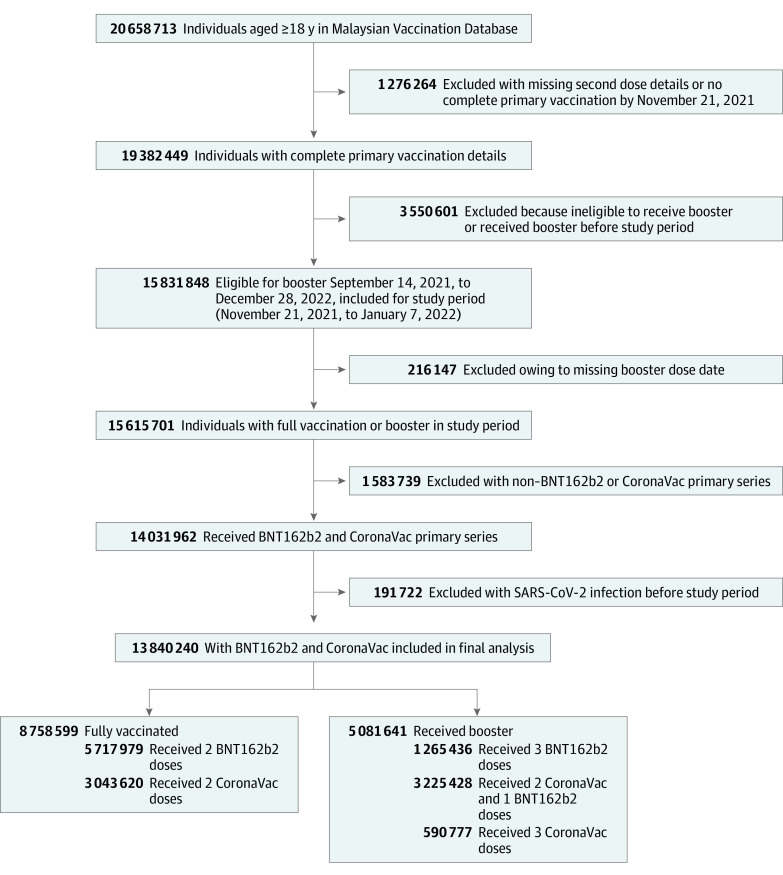
Selection Flowchart for Study Participants and Cohort Eligibility

**Figure 2.**
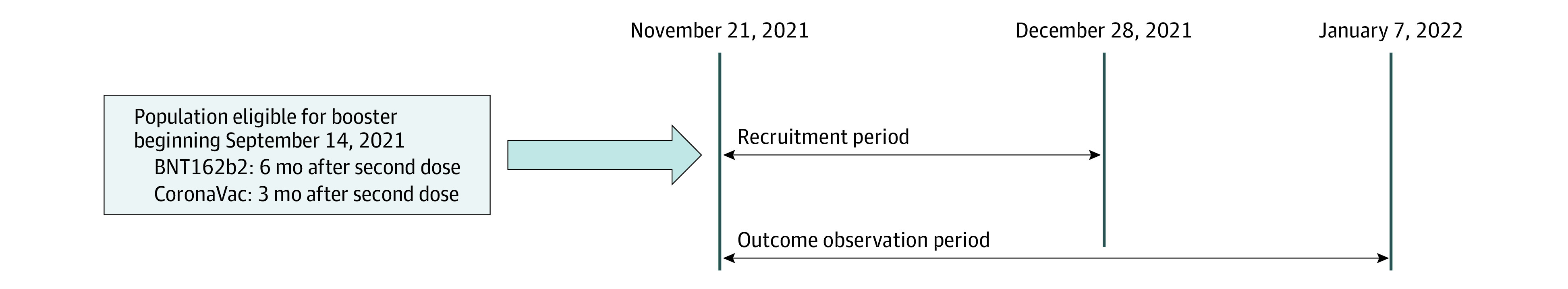
Recruitment and Observation Period

A national representative cohort among individuals aged 18 years or older who received the CoronaVac primary series and were eligible for a booster dose at 3 months after their second dose and those who received the BNT162b2 primary series and were eligible for a booster at 6 months after their second dose from November 21, 2021, to January 7, 2022, was selected for analysis (Figure 1). Individuals with documented SARS-CoV-2 infection occurring before the study period and those who received vaccines other than homologous or heterologous CoronaVac and BNT162b2 or received booster vaccines before November 21, 2021, were excluded. During the study period, only the first documented SARS-CoV-2 infection for each individual was included for analysis. Figure 1 depicts the study selection flow and the respective criteria applied. Figure 2 depicts the recruitment and observation period for the retrospective cohort studied.

### Exposure and Outcomes

Exposure was defined based on receipt of a booster dose and categorized as BNT162b2 primary series (2 doses of BNT162b2), CoronaVac primary series (2 doses of CoronaVac), 3 doses of BNT162b2, CoronaVac primary series plus BNT162b2 booster, and 3 doses of CoronaVac. Individuals were considered as fully boosted at 14 days^19^ after receiving a booster dose (booster group), whereas individuals in the nonbooster group were considered fully vaccinated at 14 days after completing their primary vaccination series of BNT162b2 or CoronaVac vaccines and did not receive any booster dose. The primary outcome included documented symptomatic SARS-CoV-2 infection by positive reverse transcription–polymerase chain reaction (RT-PCR) or rapid antigen test result. The secondary outcomes were COVID-19–related severe events, including ICU admission and death. All outcomes were observed from the day an individual was considered fully vaccinated or fully boosted until the end of study period on January 7, 2022.

### Covariates

Individual characteristics that were associated with the probability of vaccination were considered. These included a trace variable that contained the number of times an individual was in contact with an individual with COVID-19, which reflected the baseline exposure risk of the vaccine recipient, the month of achieving full vaccination to adjust for potential waning of the second dose, and vaccine purchasing mechanism to adjust for baseline behavior risk associated with socioeconomic status, mobility pattern, and health care access. Under PICK, all COVID-19 vaccines, including booster doses, were offered free of charge, but an optional opt-in private purchase mechanism was made available for CoronaVac. Variables on the presence of comorbidities (ie, chronic kidney disease, heart disease, hypertension, asthma, cancer, chronic lung disease, liver disease, stroke, immunocompromising conditions, bleeding tendency, and history of severe allergic reaction) were defined as none or 1 or more comorbidities. Frontline health care worker status and population demographics, such as age, ethnicity, state of residence, and sex, were included and adjusted in the analysis. Ethnicity was categorized as Malay, Chinese, Indians, indigenous ethnic group in Peninsular, Sabah, Sarawak, and other (referring to individuals of mixed race and individuals residing in Malaysia who were not citizens) and was based on the individual’s Malaysian identification card or passport (for non–Malaysian citizens). It was included in the analysis to adjust for potential differences in health behavior due to cultural belief.

### Statistical Analysis

The incidence rates of the outcomes (symptomatic SARS-CoV-2 infection, ICU admissions, and deaths) were calculated. Multivariable logistic regression was used to estimate the association of the different combinations of vaccines with outcomes, adjusting for aforementioned covariates. BNT162b2 primary series was chosen as the reference in the analysis to allow for comparison with other published studies. The adjusted odds ratios (aORs) were estimated for the booster combination groups (3 doses of BNT1612b2, 3 doses of CoronaVac, or 2 doses of CoronaVac plus a BNT162b2 booster) compared with the BNT162b2 primary series group for the additional protection associated with the receipt of the booster doses. In addition, we estimated the aORs in different age groups (18-39 years, 40-59 years, and ≥60 years) and within groups according to their primary vaccination coverage (BNT162b and CoronaVac subgroup). To test for sensitivity of findings from the definition of fully boosted, we separately analyzed the data using an alternative definition of fully boosted in which individuals were considered fully boosted 7 days after receiving the third dose.

All statistical analyses were conducted using SAS statistical software version 9.4 (SAS Institute). *P* values were 2-sided, and *P* < .05 was considered statistically significant. Data were analyzed from November 21, 2021, to January 7, 2022.

## Results

Our cohort included 13 840 240 individuals aged 18 years and older. The mean (SD) age was 39.9 (15.5) years, with 7 040 298 (50.9%) men and 4 451 180 individuals (32.3%) having 1 or more comorbidities; 8 758 599 individuals (63.3%) had completed only the primary vaccination series against COVID-19 and 5 081 641 individuals (36.7%) had received a booster dose. Most individuals who received the CoronaVac primary series (3 225 428 individuals [84.5%]) received a heterologous BNT162b2 booster dose. Baseline demographics and characteristics of the study cohort according to the different vaccination combination groups are provided in Table 1. Among the groups who had received a booster, the group with 3 doses of BNT162b2 had higher proportion of individuals aged 60 years and older (409 609 individuals [32.4%]) compared with those who received the CoronaVac primary series plus a BNT162b2 booster (386 511 individuals [12.0%]) or 3 doses of CoronaVac (128 483 individuals [21.8%]). Also, the group with 3 doses of BNT162b2 had a higher proportion of individuals with 1 or more comorbidities (674 291 individuals [53.3%]) compared with those who received 2 doses of CoronaVac plus a BNT162b2 booster (1 017 889 individuals [31.6%]) or 3 doses of CoronaVac (211 498 individuals [35.8%]) groups. There were 19 recipients of BNT162b2 who were recorded as private purchase, and they were excluded from subsequent analysis, as among vaccines in this study, only CoronaVac was provided under the optional private purchase program (Table 1).

**Table 1.** Baseline Characteristics of the Study Population

Characteristic	No. (%)						*P* values^a^
	All (N = 13 840 240)	Vaccine combination					
		2 Doses BNT162b2 (n = 5 714 979)	2 Doses CoronaVac (n = 3 043 620	3 Doses BNT162b2 (n = 1 265 436)	2 Doses CoronaVac + BNT162b2 booster (n = 3 225 428)	3 Doses CoronaVac (n = 590 777)	
Age, y							
Mean (SD)	39.9 (15.5)	37.8 (15.0)	36.2 (14.7)	51.4 (15.5)	41.6 (14.2)	45.6 (16.1)	<.001^b^
18-39	7 592 931 (54.9)	3 513 344 (61.5)	2 056 941 (67.6)	282 333 (22.3)	1 516 538 (47.0)	223 775 (37.9)	<.001
40-59	4 468 829 (32.3)	1 634 402 (28.6)	700 035 (23.0)	573 494 (45.3)	1 322 379 (41.0)	238 519 (40.4)	
≥60	1 778 480 (12.9)	567 233 (9.9)	286 644 (9.4)	409 609 (32.4)	386 511 (12.0)	128 483 (21.8)	
Sex							
Women	6 799 942 (49.1)	2 923 689 (51.2)	1 317 418 (43.3)	665 442 (52.6)	1 585 600 (49.2)	307 793 (52.1)	<.001
Men	7 040 298 (50.9)	2 791 290 (48.8)	1 726 202 (56.7)	599 994 (47.4)	1 639 828 (50.8)	282 984 (47.9)	
Frontline health care worker	118, 372 (0.9)	45 039 (0.8)	18 247 (0.6)	17 917 (1.4)	31 676 (1.0)	5493 (0.9)	<.001
SARS-CoV-2 infection	79 475 (0.6)	41 817 (0.7)	34 343 (1.1)	51 (<0.1)	1486 (0.1)	290 (0.1)	<.001
ICU admission	1023 (<0.1)	273 (<0.1)	713 (<0.1)	0	12 (<0.1)	3 (<0.1)	<.001
Died	508 (<0.1)	182 (<0.1)	313 (<0.1)	0	4 (<0.1)	4 (<0.1)	<.001
Comorbidities							
≥1	4 451 180 (32.2)	1 808 578 (31.7)	738 924 (24.3)	674 291 (53.3)	1 017 889 (31.6)	211 498 (35.8)	<.001
None	9 389 060 (67.8)	3 906 401 (68.4)	2 304 696 (75.7)	591 145 (46.7)	2 207 539 (68.4)	379 279 (64.2)	
Full vaccination month							
June	5344 (<0.1)	1237 (<0.1)	586 (<0.1)	1556 (0.1)	1446 (<0.1)	519 (0.1)	<.001
July	2 181 670 (15.8)	547 797 (9.6)	298 439 (9.8)	464 848 (36.7)	700 971 (21.7)	169 615 (28.7)	
August	5 360 851 (38.7)	1 636 022 (28.6)	1 301 097 (42.8)	452 882 (35.8)	1 682 886 (52.2)	287 964 (48.7)	
September	4 875 555 (35.2)	2 415 245 (42.3)	1 255 548 (41.3)	289 583 (22.9)	792 048 (24.6)	123 131 (20.8)	
October	1 416 820 (10.2)	1 114 678 (19.5)	187 950 (6.2)	56 567 (4.5)	48 077 (1.5)	9548 (1.6)	
Private purchase	200 894 (1.5)	17 (<0.1)	99 700 (3.3)	2 (<0.1)	61 233 (1.9)	39 942 (6.8)	<.001
Trace, mean (SD), No.^c^	1.55 (1.13)	1.45 (1.00)	1.62 (1.27)	1.46 (0.97)	1.64 (1.21)	1.51 (1.01)	<.001^b^

Abbreviation: ICU, intensive care unit.

^a^ Calculated using χ^2^ unless otherwise noted.

^b^ Caclulated using F test.

^c^ Defined as the number of times an individual was in contact with an individual with COVID-19.

Table 2 shows the number of events and unadjusted incidence rates per 100 000 individuals of symptomatic SARS-CoV-2 infection, ICU admission, and deaths for the different vaccine combination groups. For all 3 outcomes, individuals with 3 doses of BNT162b2 had the lowest number of events and unadjusted incidence rates. For symptomatic SARS-CoV-2 infection, 51 infections were observed for individuals with 3 doses of BNT162b2 (4.03 events per 100 000 individuals), compared with 1486 infections among those with 2 doses of CoronaVac plus a BNT162b2 booster (46.07 events per 100 000 individuals) and 290 infections among those with 3 doses of CoronaVac (49.09 events per 100 000 individuals). There were no ICU admissions or deaths observed among the group with 3 doses of BNT162b2 during the study period. For both of these outcomes, the unadjusted incidence rates were higher for those with 3 doses of CoronaVac (1.02 ICU admissions per 100 000 individuals; 0.51 deaths per 100 000 individuals) compared with 2 doses of CoronaVac plus a BNT162b2 booster (0.37 ICU admissions per 100 000 individuals; 0.12 deaths per 100 000 individuals).

**Table 2.** Infection, ICU Admission, and Death Rate of the Cohort

Vaccination group	Total, No.	Events, No. (rate per 100 000 individuals)		
		Infections	ICU admissions	Deaths
2 Doses BNT162b2	5 714 962	41 817 (731.71)	273 (4.78)	182 (3.18)
2 Doses CoronaVac	3 043 620	34 343 (1128.36)	713 (23.43)	313 (10.28)
3 Doses BNT162b2	1 265 434	51 (4.03)	0 (NA)	0 (NA)
2 Doses CoronaVac + BNT162b2 booster	3 225 428	1486 (46.07)	12 (0.37)	4 (0.12)
3 Doses CoronaVac	590 777	290 (49.09)	6 (1.02)	3 (0.51)

Abbreviations: ICU, intensive care unit; NA, not applicable.

Table 3 shows the unadjusted and adjusted ORs of SARS-CoV-2 infection by vaccine combination and age groups. Compared with individuals who received the BNT162b2 primary series, the adjusted odds of symptomatic SARS-CoV-2 infection were lowest for individuals who received 3 doses of BNT162b2 (aOR, 0.01 [95% CI, 0.00-0.01]), followed by those who received the CoronaVac primary series plus a BNT162b2 booster (aOR, 0.06 [95% CI, 0.05-0.06]) and those who received 3 doses of CoronaVac (aOR, 0.08 [95% CI, 0.06-0.10]). As for the COVID-19–related severe outcomes, because the observed events for these outcomes were very small among the boosted groups, we were unable to reliably estimate the measures of association.

**Table 3.** Unadjusted and Adjusted Regression Analysis of Protection Against Symptomatic SARS-CoV-2 Infection

Vaccination group	Full cohort^a^		Adjusted OR by age group (95% CI)^a^^,^^b^		
	OR (95% CI)	Adjusted OR (95% CI)^b^	18-39 y	40-59 y	≥60 y
2 Doses CoronaVac	1.55 (1.53-1.57)	1.76 (1.71-1.82)	1.76 (1.69-1.83)	1.92 (1.80-2.05)	3.96 (3.95-3.97)
3 Doses BNT162b2	0.01 (0.00-0.01)	0.01 (0.00-0.01)	0.01 (0.01-0.02)	0.00 (0.00-0.01)	0.10 (0.14 0.14)
2 Doses CoronaVac + BNT162b2	0.06 (0.06-0.07)	0.06 (0.05-0.06)	0.05 (0.04-0.05)	0.07 (0.06-0.08)	0.17 (0.17-0.18)
3 Doses CoronaVac	0.07 (0.06-0.08)	0.08 (0.06-0.10)	0.08 (0.06-0.12)	0.08 (0.06-0.12)	0.19 (0.19-0.20)

Abbreviation: OR, odds ratio.

^a^ Comparisons calculated with the 2 doses BNT162b2 group as the reference.

^b^ Adjusted for age, ethnicity, sex, state, baseline exposure risk (trace), frontline health care worker status, month of full vaccination, procurement mechanism, and presence of comorbidities.

The CoronaVac nonbooster group was observed to have 76% higher adjusted odds of symptomatic SARS-CoV-2 infection compared with the primary series BNT162b2 group (aOR, 1.76 [95% CI, 1.71-1.82]). Among individuals aged 60 years and older, the associated odds were almost 4-fold higher (aOR, 3.96 [95% CI, 3.95-3.97]). Receipt of a booster dose, either a heterologous (BNT162b2) or homologous (CoronaVac), was associated with a reduction of more than 90% for odds against symptomatic SARS-CoV-2 infection (≥80% among individuals aged ≥60 years). Among individuals aged 60 years and older, CoronaVac primary series plus a BNT162b2 booster was associated with a lower adjusted risk of symptomatic SARS-CoV-2 infection (aOR, 0.17 [95% CI, 0.17-0.18]) than 3 doses of CoronaVac (aOR, 0.19 [95% CI, 0.19-0.20]).

Subgroup analysis within the CoronaVac primary series recipients was also consistent observing that the boosted groups (either heterologous or homologous) were associated with more than 90% reduction in odds against symptomatic SARS-CoV-2 infection compared with the CoronaVac nonbooster group (eTable 1 in the Supplement). A sensitivity analysis was performed using an alternative cutoff of 7 days after receiving booster dose (eTable 2 in the Supplement). The measures of association (aORs) estimated using this alternative cutoff of 7 days after booster receipt were consistent with that of 14 days after booster receipt used in main analysis (Table 3), albeit lower across all booster combinations for all age groups.

## Discussion

In this retrospective cohort study using nationally representative data sets, we found that the administration of a booster dose, either homologous (CoronaVac) or heterologous (BNT162b2), at 3 months after completion of the CoronaVac primary series (2 doses), was similar in the estimated odds against of symptomatic SARS-CoV-2 infection to homologous (BNT162b2) boosting at 6 months interval for the BNT162b2 primary series (2 dose) among all adult age groups. For severe outcomes of ICU admission and deaths, both homologous and heterologous boosted CoronaVac groups were observed to have higher rates compared with homologous boosted BNT162b2; nevertheless, the rates were substantially lower compared with nonboosted groups of either vaccine. Our study findings provide evidence to support a flexible approach to boosting for all adult recipients of the CoronaVac primary series at 3 months after primary vaccination.

In our study, the adjusted odds of symptomatic SARS-CoV-2 infection for CoronaVac primary series was 76% higher compared with the BNT162b2 primary series, and escalating to almost 4-fold higher for individuals aged 60 years and older. This is consistent with our previous findings of greater waning observed among recipients of CoronaVac^18^ and supports the consideration for booster dose administration, especially among older adults, as recommended by the WHO,^15^ as older adults mount a lower immune response following a standard primary series of inactivated vaccines, such as CoronaVac.

While both homologous and heterologous booster regimens are immunologically effective,^20^ immunogenicity studies reported higher levels of SARS-CoV-2–specific antibodies, including neutralizing antibodies, in heterologous boosting groups.^11,21^ In a study conducted in Chile, heterologous boosting with BNT162b2 yielded better adjusted vaccine effectiveness (VE) estimates than homologous boosting with CoronaVac for all outcomes under investigation (symptomatic infection, hospitalization, ICU admission, and COVID-19–related deaths).^22^ Our study findings were consistent in observing that both regimens were associated with protection against symptomatic SARS-CoV-2 infection and at higher estimates for heterologous boosting among individuals aged 60 years and older.

Immunogenicity studies have shown that antibody levels wane within 3 to 6 months after the second dose completion.^11,13^ In a study in Brazil by Cerqueira-Silva et al,^23^ heterologous boosting with BNT162b2 at 6 months after the second dose of CoronaVac was associated with a reduction of 92.7% for risk of infection and 97.3% for risk of severe outcomes. In another study conducted in Chile,^22^ homologous boosting with CoronaVac and heterologous boosting with BNT162b2 at 4-month intervals after CoronaVac primary series were associated with a reduction of 78.8% and 96.5% in risk of infection, respectively. Our study findings add to the body of evidence by reporting the aOR estimates against symptomatic SARS-CoV-2 infection for both homologous and heterologous booster regimens given after 3 months interval following full vaccination with CoronaVac. The findings provide valuable information to policy makers in other countries where CoronaVac is used in their national vaccination program.

In our study, we also demonstrated that homologous boosting for BNT162b2 primary series recipients was associated with more than 99% reduction in odds of symptomatic SARS-CoV-2 infection, with no observed severe outcomes over the short study period. This is in line with evidence from other studies, where homologous triple BNT162b2 vaccinations has been shown to protect against similar outcomes.^2,3,6,24^ The variation in the observed measures of association estimates with other studies may be owing to the relatively short outcomes observation period for this study.

Our study draws strength from the use of consolidated national cohort databases of all vaccinated individuals in Malaysia, from which data from more than 13.8 million individuals were used to derive study findings. During this study period, Malaysia had a background of more than 4000 daily confirmed SARS-CoV-2 infections (range, 2589 to 6144 cases), predominantly of the Delta variant.

### Limitations

Our study has limitations. First, our findings were limited by the study outcomes observation period. Hence, we were unable to provide reliable association estimates for severe outcomes. The long-term benefit of a booster vaccination program will require a longer follow-up period to establish stable estimates of protection and the duration of protection. Second, our study period also largely preceded the emergence of the Omicron variant of SARS-CoV-2 in Malaysia. Although the first detected case of the Omicron SARS-CoV-2 variant in Malaysia was reported on December 3, 2021, there was no significant surge Omicron cases until the end of the study period,^25^ suggesting that most SARS-CoV-2 infections in this study are likely be the Delta variant. Therefore, findings from our study need to be interpreted with caution, since recent studies^19,26,27^ have observed lower vaccine-derived protection from the administration of booster vaccinations against the Omicron variant. Third, symptomatic SARS-CoV-2 infection was used as an outcome because during the study period, Malaysia adopted a national testing strategy in which supervised testing using RT-PCR or antigen tests was administered for close contacts and individuals who were symptomatic. Nevertheless, asymptomatic SARS-CoV-2 infection may still be included in our analysis, as there were no formal restrictions to supervised testing. Fourth, although we adjusted for the potential confounders that could affect the probability of getting a booster dose, we were unable to fully adjust for unobservable or unmeasurable confounders, such as the social risk for infection between the study groups and adherence to nonpharmaceutical interventions. Furthermore, although our study findings have important implications since CoronaVac is widely used in many low- and middle-income countries globally,^28^ the generalizability may be limited by differences in vaccine rollout and coverage, population demographics, and the background COVID-19 situation, including the COVID-19 case burden and the predominant circulating COVID-19 variants.

## Conclusions

This cohort study found that flexible boosting (heterologous and homologous) at 3 months for recipients of the CoronaVac primary series was similar to homologous boosting of BNT162b2 at 6 months for recipients of the BNT162b2 primary series for odds against symptomatic SARS-CoV-2 infection. Among the adults aged 60 years and older, heterologous boosting using BNT162b2 booster for recipients of the CoronaVac primary series should take priority, given the lower observed odds against symptomatic SARS-CoV-2 infection.

## Notes

Supplement. **eTable 1.** Subgroup Analysis With Primary Vaccination Series as the Reference Group **eTable 2.** Unadjusted and Adjusted Odds Ratios for Risk of Symptomatic SARS-CoV-2 Infection Using 7 Days After Third Dose as Fully Boosted
