# Supplementary material for: Assessment of Heterologous and Homologous Boosting With Inactivated COVID-19 Vaccine at 3 Months Compared With Homologous Boosting of BNT162b2 at 6 Months
Source: JAMA Netw Open. 2022 Aug 10;5(8):e2226046. doi: 10.1001/jamanetworkopen.2022.26046 (PMC9366545; doi:10.1001/jamanetworkopen.2022.26046)
Supplement: Supplement. — eTable 1. Subgroup Analysis With Primary Vaccination Series as the Reference Group eTable 2. Unadjusted and Adjusted Odds Ratios for Risk of Symptomatic SARS-CoV-2 Infection Using 7 Days After Third Dose as Fully Boosted [file jamanetwopen-e2226046-s001.pdf]

Supplemental Online Content

Low EV, Tok PSK, Husin M, et al. Assessment of heterologous and homologous boosting with inactivated COVID-19 vaccine at 3 months compared with homologous boosting of BNT162b2 at 6 Months. *JAMA Netw Open*. 2022;5(8):e2226046. doi:10.1001/jamanetworkopen.2022.26046

eTable 1. Subgroup Analysis With Primary Vaccination Series as the Reference Group

eTable 2. Unadjusted and Adjusted Odds Ratios for Risk of Symptomatic SARS-CoV-2 Infection Using 7 Days After Third Dose as Fully Boosted

This supplemental material has been provided by the authors to give readers additional information about their work.

© 2022 Low EV et al. *JAMA Network Open.*

eTable 1: Subgroup Analysis using primary vaccination series as reference group

Type of Vaccines Combination	Adjusted OR (95% CI)[a]
BNT162b2 as Primary series	
3 Doses of BNT162b2 vs. 2 doses of BNT162b2	0.01 (0.00, 0.01)
CoronaVac as Primary series	
2 Doses CoronaVac + BNT162b2 vs. 2 doses of CoronaVac	0.03 (0.03, 0.04)
3 Doses of CoronaVac vs. 2 dose of CoronaVac	0.04 (0.03, 0.05)

[a]Adjusted for age, ethnicities, sex, state, health care worker status, baseline exposure risk (trace), month of full vaccination, procurement mechanism, and presence of comorbidities

© 2022 Low EV et al. *JAMA Network Open.*

eTable 2: Unadjusted and Adjusted Odds Ratio Against Symptomatic SARS-CoV-2 Infection using fully booster as 7 days after third dose

Type of Vaccines Combination[a]	Full cohort		18 to 39 years old	40 to 59 years old	60 years old and above
	OR (95% CI)	Adjusted OR (95% CI)[a]	Adjusted OR (95% CI)[a]	Adjusted OR (95% CI)[a]	Adjusted OR(95% CI)[a]
2 Doses of CoronaVac vs. 2 Doses of BNT162b2	1.55 (1.53, 1.57)	1.76 (1.71, 1.82)	1.76 (1.69, 1.82)	1.93 (1.80, 2.06)	3.66 (3.66, 3.67)
3 doses of BNT162b2 vs. 2 doses of BNT162b2	0.01 (0.01, 0.01)	0.01 (0.01, 0.02)	0.02 (0.01, 0.03)	0.01 (0.00, 0.01)	0.15 (0.15, 0.15)
2 Doses CoronaVac + BNT162b2 vs. 2 doses of BNT162b2	0.11 (0.11, 0.12)	0.10 (0.09, 0.11)	0.09 (0.08, 0.10)	0.12 (0.11, 0.13)	0.20 (0.20, 0.20)
3 Doses of CoronaVac vs. 2 dose of BNT162b2	0.13 (0.12, 0.14)	0.15 (0.13, 0.18)	0.17 (0.14, 0.22)	0.15 (0.12, 0.20)	0.24 (0.23, 0.24)

[a]Adjusted for age, ethnicities, sex, state, baseline exposure risk (trace), frontliner status, month of full vaccination, procurement mechanism, and presence of comorbidities

© 2022 Low EV et al. *JAMA Network Open.*
